# Amino terminal recognition by a CCR6 chemokine receptor antibody blocks CCL20 signaling and IL-17 expression via β-arrestin

**DOI:** 10.1186/s12896-021-00699-2

**Published:** 2021-07-05

**Authors:** Sara Gómez-Melero, Fé Isabel García-Maceira, Tania García-Maceira, Verónica Luna-Guerrero, Gracia Montero-Peñalvo, Isaac Túnez-Fiñana, Elier Paz-Rojas

**Affiliations:** 1 Canvax Biotech, Parque Científico y Tecnológico Rabanales 21, c/Astrónoma Cecilia Payne s/n, Edificio Canvax, 14014 Córdoba, Spain; 2 Department of Biochemistry and Molecular Biology, School of Medicine, University of Córdoba, Avda. Menéndez Pidal s/n, 14004 Córdoba, Spain

**Keywords:** Th17 cells, CCR6, Inflammation, Therapeutic antibody, GPCR, Bias signaling

## Abstract

**Background:**

CCR6 chemokine receptor is an important target in inflammatory diseases. Th17 cells express CCR6 and a number of inflammatory cytokines, including IL-17 and IL-22, which are involved in the propagation of inflammatory immune responses. CCR6 antagonist would be a potential treatment for inflammatory diseases such as psoriasis or rheumatoid arthritis. The aim of this study is to develop an antagonistic monoclonal antibody (mAb) against human CCR6 receptor (hCCR6).

**Results:**

We generate monoclonal antibodies against hCCR6 immunizing Balb/c mice with hCCR6 overexpressing cells. The antibodies were tested by flow cytometry for specific binding to hCCR6, cloned by limiting dilution and resulted in the isolation and purification monoclonal antibody 1C6. By ELISA and flow cytometry, was determined that the antibody obtained binds to hCCR6 N-terminal domain. The ability of 1C6 to neutralize hCCR6 signaling was tested and we determined that 1C6 antibody were able to block response in β-arrestin recruitment assay with IC_50_ 10.23 nM, but did not inhibit calcium mobilization. In addition, we found in a chemotaxis assay that 1C6 reduces the migration of hCCR6 cells to their ligand CCL20. Finally, we determined by RT-qPCR that the expression of IL-17A in Th17 cells treated with 1C6 was inhibited.

**Conclusions:**

In the present study, we applied whole cell immunization for successfully obtain an antibody that is capable to neutralize hCCR6 signaling and to reduce hCCR6 cells migration and IL-17 expression. These results provide an efficient approach to obtain therapeutic potential antibodies in the treatment of CCR6-mediated inflammatory diseases.

**Supplementary Information:**

The online version contains supplementary material available at 10.1186/s12896-021-00699-2.

## Background

G-protein-coupled receptors (GPCRs) represent 4% of the human genome and are one of the most attractive therapeutic targets due to mediate many important physiological functions [1]. These receptors are characterized by seven transmembrane helices with three intracellular loops, three extracellular loops, an extracellular N-terminal domain and an intracellular C-terminus. The human CC chemokine receptor 6, hCCR6, is a class A of GPCR expressed in a particular diverse range of leukocytes including T cells (specifically Th17 cells and Treg cells), B cell, neutrophils and subsets of dendritic cells [2].

Chemokine–chemokine receptor interaction supports numerous inflammatory, autoimmune conditions and metastatic process in many cancers. In inflammatory diseases, the critical function of hCCR6 is maintaining leukocyte homeostasis through the recruitment of Th17 and Treg cells to sites of inflammation [3]. The only known endogenous chemotactic ligand for hCCR6 is CC chemokine ligand 20 (CCL20) which is produced by Th17 cells. The axis hCCR6/CCL20 influences in the respiratory, gastrointestinal, skeletal, and reproductive systems through pleiotropic immune mechanisms. Some studies indicated that upon CCR6-CCL20 binding is activated a myriad of signaling pathway through heterotrimeric G protein (αi), calcium mobilization, PLC-β, phosphatidylinositol 3-kinase/Akt, ERK1/2 phosphorylation, actin polymerization, and beta arrestins [4]. Of particular relevance in rheumatoid arthritis, psoriasis, multiple sclerosis, inflammatory bowel disease and cancer; inhibition of the hCCR6 signaling might prove to be a useful therapeutic strategy [2, 5].

Despite being one of the largest classes of proteins, the majority of GPCRs remains undragged, with only approximately 100 GPCRs targeted with small molecules or peptides. Antibodies against GPCRs offer an alternative to conventional small molecule drugs, that are often unsuccessful, and could provide valuable new treatment options [6]. Nevertheless, to date, there is no therapeutic antibody against hCCR6 approved [7] and develop an antagonizing monoclonal antibody (mAb) should be a potential alternative to conventional small molecule drugs and an effective strategy for the treatment of certain inflammatory and autoimmune diseases.

The generation of relevant antibodies against GPCRs with the required specificity and functionality remains a challenge. A critical factor is obtaining sufficient amounts of functional antigen in biologically relevant formats. GPCRs often expressed at low levels in cells and are very unstable when purified. The ideal antigen format would be homogenous, stable and must contain the relevant post translational modifications. Usually, antibodies obtained have no effect on receptor function and have limited utility as therapeutic agents [7–9].

In the present study, we used immunization with whole cells overexpressing hCCR6 receptor to establish a monoclonal antibody against hCCR6. Antibodies that bind human CCR6 were identified and, the best antibody obtained, was selected for further detailed in vitro characterization which is described here. We successfully obtained a monoclonal antibody antagonistic of β-arrestin signaling that recognizes the N-terminal domain, inhibits the migration of hCCR6 cells to CCL20 and reduces the expression of IL-17A on Th17 cells.

## Materials and methods

### Plasmids

Coding sequences of human CCR6 (Gb: NM_004367), mouse CCR6 (Gb: NM_001190333), human CCR1 (Gb: NM_001295), human CCR3 (Gb: NM_001837), human CCR4 (Gb: NM_005508), human CCR5 (Gb: NM_000579), human CXC1R (Gb: NM_000634), human CXC2R (Gb: NM_001557), human CXC3R (Gb: NM_001504) and human CXC4R (Gb: NM003467)) were obtained by synthesis flanked with the recognition sites of 5’*Xho*I and 3′*Not*I. They were cloned in frame with the signal peptide IgΚ, a short sequence to improve GPCR expression [10], and a TAG sequences (c-myc or HA) into the pcDNA3.1 vector by the restriction with the indicated enzymes.

Construction of chimeric receptors was carried out by overlap extension polymerase chain reaction using human CCR6 or murine CCR6 vectors. The integrity of the constructs was confirmed by sequence analysis (Stabvida).

### Cell culture and transfection

All cell lines were purchased from the American Type Culture Collection (ATCC). P815 (murine mastocytoma, ATCC TIB-64) cells were cultured in Dulbecco’s Modified Eagle Medium (DMEM), RBL-2H3 (rat basophilic leukemia, CRL-2256) in Eagle’s minimum essential *medium* (EMEM)/RPMI-1640 medium and NS1 (murine myeloma cell, ATCC TIB-18) in RPMI-1640 medium. Hybridoma cells were maintained in RPMI-1640 medium. All media (Gibco) were supplemented with 10% fetal bovine serum (FBS*,* Gibco), 2 mM l-glutamine (Lonza), 100 U/mL penicillin (Gibco), and 100 μg/mL streptomycin (Gibco). Cells were maintained at 37 °C in a humidified atmosphere of 5% CO_2_.

RBL and P815 cells were transfected with the expression vectors obtained using Canfast (Canvax Biotech) according to the manufacturer’s instructions. Following transfection, stably transfectans were cultured with 0.5 mg/mL G418 (Phytotechnology). The GPCRs expression levels were determined using FACSCalibur flow cytometer (Becton Dickinson). To confirm the plasmid transfection, we used flow cytometry against c-myc (EQKLISEEDL) tag or HA tag (YPYDVPDYA), using anti-HA FITC (Miltenyi Biotech) and mouse anti c-myc (9E10, in house) antibodies, respectively.

### Animals

Balb/C mice were used to the immunization program. Animal were housed in the Animal Experimentation Unit of Cordoba University (Cordoba, Spain) under standard colony conditions: 12 h: 12 h light: darkness cycle (lights on at 7:00 a.m.), controlled room temperature (22 ± 2 °C), with free access to food and water.

This study was carried out according to the guidelines of the Directive of 24 November 1986 (86/609/ECC) approved by the European Communities Council and RD 53/2013 passed by the Presidency Minister of Spain (BOE, 8 February, 2013). The protocols were approved by the Bioethics Committee of the Junta de Andalucía with the number 09/7/15/280.

### Immunization and hybridoma generation

To establish an anti-hCCR6 mAb, female BALB/c mice of 8 weeks old were immunized with P815 cells c-myc tagged overexpressing the human CCR6 receptor. For immunizations, P815-c-myc-hCCR6 cells were harvested and incubated with 10 μg/mL of mitomycin C (Sigma) for 60 min at 37 °C and then washed three times and resuspended in phosphate-buffered saline (PBS). Each mouse was injected intraperitoneally with 10^7^ mitomycin C-treated cells six times at 2-week intervals, followed by three injections once a month. One week after the last boost, sera were collected by centrifugation and titers for specific antibodies were determined by cell membrane ELISA. Selected mouse was boosted intravenously with 10^7^ cells 4 days prior to sacrifice. Collected splenocytes were fused to NS1 mouse myeloma cells using the polyethylene glycol (Sigma) fusion method [11]. Two weeks post-fusion, culture supernatants were screened by flow cytometry for hCCR6-specific antibodies using RBL-HA-hCCR6 cells to remove the clones that reacted with c-myc and with P815 cells. Positive hybridomas were subcloned by limiting dilution.

### Flow cytometry

For the screening of hybridoma cells and characterization of purified mAbs against hCCR6 flow cytometric analysis was performed. Undiluted hybridoma supernatants or different concentrations of purified mAbs were incubated with RBL cells stably expressing HA-hCCR6 or mock transfectants for 20 min at 4 °C. After washing with PBS, cells were stained with FITC-conjugated goat anti-mouse IgG (1:60, Sigma) for 10 min at 4 °C and were analyzed by flow cytometry using a FACSCalibur (Becton Dickinson). A total of 10,000 events were acquired and data were analyzed with BD CellQuest Pro software (BD Biosciences).

### Anti-hCCR6 control antibodies

The sequences of the variable domains of the anti hCCR6 control positive, KM4703 (WO/2013/005649, [12]), were retrieved from the patent literature available in open access. The V_H_ and V_L_ domains corresponding sequences were synthesized by Integrated DNA Technologies (IDT) and they were fused in frame with the mouse heavy chain constant domains or mouse light chain (kappa) constant domains together with elements to express whole IgG heavy chain in mammalian cells. Heavy and light chain IgG expressing vectors were transfected into ExpiCHO cell line (Gibco) and the antibody was purified of culture supernatant. Anti-NGFR (HB8737, in house) was used as an isotype control antibody.

### Antibody purification

Immunoglobulins were affinity-purified from the culture supernatant using Protein G Sepharose columns (HiTrap Protein G HP, GE Healthcare) according to the manufacturer’s protocol in AKTA Prime Plus (GE Healthcare). Affinity-purified mAbs were separated by SDS-PAGE on 10% acrylamide gels and stained with Coomassie brilliant blue. The concentration of purified immunoglobulins was determined by UV absorbance at 280 nm. The isotype of the 1C6 antibody was determined using a mouse mAb isotyping kit (SinoBiological) according to the manufacturer’s instructions.

### Cell membrane extraction

To extract the membrane fractions, cell pellets (2 × 10^7^ cells) from RBL-HA-hCCR6 or mock transfectans were resuspended in 4 mL of lysis buffer (Sucrose 250 mM, HEPES 4 mM, EDTA 1.5 mM, pH 7,4) with protease inhibitors (PMSF 1 mM, leupeptin 1 μg/mL, pepstatin 1 μg/mL, aprotinin 1 μg/mL). The cells were then transferred into Dounce homogenizer and 15–20 strokes were applied to produce cell lysates. Unbroken cell, nuclei and cell debris were removed by centrifuging the lysates twice at 800 g for 10 min. The supernatants were then centrifuged at 1000 g for 10 min. Finally, the supernatant obtained was centrifuged at 15,000 g for 25 min. The membrane pellet was resuspended in PBS and protein concentration was determined by the Bradford assay (Bio-Rad). All fractionation steps were carried out at 4 °C.

## ELISA

Sera from immunized mice and binding of purified mAbs to hCCR6 N-terminal region were analyzed by ELISA. To test the serum titer, cell membrane solutions were coated directly at 10 μg/mL in buffer coating (Na_2_CO_3_ and NaHCO_3,_ 0.1 M, pH 9.6) onto 96-well maxisorp plates (Thermo) overnight at 4 °C. To asses binding of mAb against to the hCCR6 N-terminal region, a peptide with the N-terminal fragment of hCCR6 (aa M1–L47, Canvax Biotech) was coated at 1 μg/mL in buffer coating onto a 96-well plate overnight at 4 °C. Then, the plate was washed 3 times with PBS containing 0.05% Tween-20 and blocked for 1 h at 37 °C with PBS plus 5% bovine serum albumin (BSA). Following this, the plate was incubated for 2 h with serially diluted serum or 0.5 μg/mL of purified mAbs in PBS-0.5% BSA. After washing with PBS-0.05% Tween 20, the plate was incubated for 1 h at 37 °C with a 1:8000 dilution of horseradish-peroxidase (HRP)-conjugated goat anti-mouse IgG antibody (Sigma). After final washes, bound IgG was detected using the 3,3′,5,5′-tetramethylbenzidine (TMB) liquid substrate system (Medicago). The absorbance was measured at 450 nm using a FLUOStar OPTIMA plate reader (BMG Labtech).

The concentration of CCL20 in conditioned media was measured using a specific Human CCL20/MIP-3 Alpha Duoset ELISA (R&D Systems) following the manufacturer’s instructions.

### Epitope mapping

RBL HA tagged cells expressing the first, second and third extra-cellular loops, (ECL1, ECL2, and ECL3) and N-terminal region of hCCR6 embedded into mCCR6 sequence were obtained and used in a standard flow cytometry to elucidate epitope mapping of the mAb 1C6. The cells were stained with 1 μg/mL of hCCR6 antibody 1C6 followed by goat anti-mouse IgG FITC antibody (Sigma). Interaction between mAb and swapped mutants was analyzed by flow cytometry. To validate expression of swapped mutants, mouse anti-HA FITC antibody (Miltenyi Biotech, 1:10) was used.

### Cross-reactivity

Stably transfected RBL HA tagged cells with pcDNA3.1 plasmid comprising synthetic gene of human CCR1, CCR3, CCR4, CCR5, CXC1R, CXC2R, CXC3R and CXC4R receptors were obtained. To validate expression of chemokine receptors on transfected cells, mouse anti-HA FITC antibody (Miltenyi Biotech, 1:10) was used. These cell lines were labeled with the hCCR6 antibody obtained to evaluate their specificity by flow cytometry.

### Calcium flux assay

To asses antagonistic activity of anti-hCCR6 1C6 antibody in calcium mobilization we used FRIDA assay (WO/2012/013204, [13]). Mobilization of Ca^2+^ was determined using RBL-hCCR6 cells. The cells were plated in 384-well plates (Corning, 384-well flat-bottom black polystyrene microplates) at 5000 cells per well and cultured for 48 h at 37 °C, 5% CO_2_. Then, cell media was aspirated and replaced with 20 μL anti-hCCR6 antibodies diluted in BSS (25 mM HEPES/NaOH, 1.2 mM KH_2_PO_4_, 65 mM NaCl, 5.65 mM KCl, 0.6 mM MgCl_2_, 1.8 mM CaCl_2_, 5.6 mM Glucose and 0.1% BSA) per well. Cells were incubated 15 min at 37 °C, 5% CO_2_. Then, the cells were incubated at 37 °C for 60 min with CCL20 (Peprotech) ligand at EC_85_ concentration (10 nM) and 1 mM 4-Mthylumbelliferyl N-acetyl-β-d-glucosaminide substrate (Glycosynth) in BSS protected from light. The fluorescence was measure (excitation: 360 nm, emission: 470 nm) using a FLUOStar OPTIMA plate reader (BMG Labtech). Data was expressed as normalized percent inhibition (NPI) in which the difference between the sample measurement and the mean of the positive controls (ligand at EC_85_ without antibody) is divided by the difference between the means of the measurements on the positive and the negative controls (without ligand).

### β-Arrestin recruitment assay

The PathHunter™ protein complementation assay (DiscoveRx Corporation) was performed according to the manufacturer’s instructions with minor modifications to evaluate antagonistic activity of anti-hCCR6 1C6 antibody in β-arrestin recruitment. The cells were plated in 384-well plates (Greiner bio-one, 384-well flat-bottom white polypropylene microplates) at 7000 cells per well and cultured for 48 h at 37 °C, 5% CO_2_. Next, the cells were incubated at 37 °C for 90 min with anti-hCCR6 antibodies and CCL20 (Peprotech) ligand at EC_85_ concentration (10 nM) in assay media. Gal-Screen substrate (Applied Biosciences) was then added and incubated at 23 °C protected from light for 60 min before measuring luminescence using a plate reader (Perkin Elmer EnVision). The normalized percent inhibition (NPI) was calculated as described above and the IC_50_ value was generated using GraphPad Prism software using a model ‘log [inhibitor] vs. response’.

### Chemotaxis assay

Chemotaxis was assayed using 6.5 mm transwell tissue culture polycarbonate inserts with 8 μm pores (Corning). For the assay, RBL-2H3 cells stably expressing hCCR6 were washed once with PBS and, for inhibition assay, incubated with 1C6 antibody for 30 min. Then, cells were washed with PBS, resuspended at a density of 10^6^ cells/mL in assay buffer (DMEM containing 2 mM glutamine and 0.2% BSA) and two hundred of cells were added into the upper chamber of transwell which was contained in a well with 600 μL of assay buffer with or without 125 nM of human CCL20. The assay was incubated for 22 h at 37 °C (5% CO_2_). Cells that migrated to the lower chamber were collected and stained with 8 μM of calcein AM (Santa Cruz Biotechnology) and the fluorescence signal was measured (excitation: 485 nm, emission: 530 nm) using a FLUOStar OPTIMA plate reader (BMG Labtech). The chemotactic index was calculated by dividing the number of migrated cells to CCL20 by that in absence of chemokine (background).

### Human T cells isolation, culture and Th17 cells differentiation

Buffy coats were obtained from healthy human donors (Hospital Reina Sofia, Córdoba) with fully informed consent, conducted in accordance with ethical standards of the Declaration of Helsinki and approved by the Ethics Committee of the Hospital Reina Sofia (CEIC4603/310/30062020).

Human peripheral blood mononuclear cells (PBMC) were purified by density gradient centrifugation using Histopaque-1077 (Sigma) for 30 min at 400 g, at room temperature. T cells were isolated by negative selection using Pan T Cell Isolation Kit human (Miltenyi Biotec) and were stimulated via CD3/CD28 (Life technologies) at 1:2 bead to cell ratio. Cells were culture at 37 °C in a 5% CO_2_ humidified atmosphere in X-VIVO 15 (Lonza) medium supplemented with 10% FBS and, for Th17 polarizing conditions, 20 ng/mL IL-1β (Peprotech), 30 ng/mL IL-6 (Peprotech), 30 ng/mL IL-23 (Peprotech), 2.25 ng/mL TGF-β1 (Peprotech) and 2.5 μg/mL anti-IL4 (Mabtech) were added. After 8 days, cells were plated at 5 × 10^5^ cells/mL in media containing 5 ng/mL IL-1β, 7.5 ng/mL IL-6, 7.5 ng/mL IL-23, 0.56 ng/mL TGF-β1 and 0.625 μg/mL anti-IL4 or in absence of polarizing conditions for control cells and with or without 62 nM of anti-CCR6 antibodies for additional 5 days. Then, cells were collected for total RNA isolation and supernatants were collected and stored at − 80 °C for further cytokine measurement.

### Quantitative real-time PCR

RNA was extracted from Th17 cells o control T cells according to the manufacturer’s protocol for PRImeZOL reagent (Canvax Biotech). Moloney murine leukemia virus reverse transcriptase (Canvax Biotech) was used to synthetize cDNA from the total RNA using reverse primers. Quantitative Real-Time PCR assay (qRT-PCR) for measurement IL-17A and β-actin mRNA expression was performed using StepOne Real-Time PCR System (Applied Biosystems). The following primers and probes sequences were used (5′-3′): IL-17A forward TGGGAAGACCTCATTGGTGT, IL-17A reverse GGATTTCGTGGGATTGTGAT, IL-17A probe FAM-CTGCTGCTGAGCCTGGAG-BHQ1, β-actin forward GAAACTACCTTCAACTCCATC, β-actin reverse CTTGCTGATCCACATCTGCTG and β-actin probe FAM-ACCCAGCACAATGAAGATCAAGATCAT-TAMRA. Relative RNA levels in the samples were determined using standard curves prepared from five-fold serial dilutions of cDNA from the pool of the samples. Relative expression levels of gene were normalized to β-actin in the samples and quantification was performed using the 2^-ΔΔCt^ method. All samples were measured in duplicate and the average value of both duplicates was used as the quantitative value.

### Statistical analysis

The statistical analysis was carried out using GraphPad Prism version 8.0.1 software (GraphPad Software). The statistical differences for the mean values were analyzed using one way ANOVA with Tukey test and are indicated with *, *p* < 0.05; **, *p* < 0.01; ***, *p* < 0.001; and ****, *p* < 0.0001.

## Results

### Generation of monoclonal antibodies to human CCR6 by immunization with cells

**Fig. 1:**
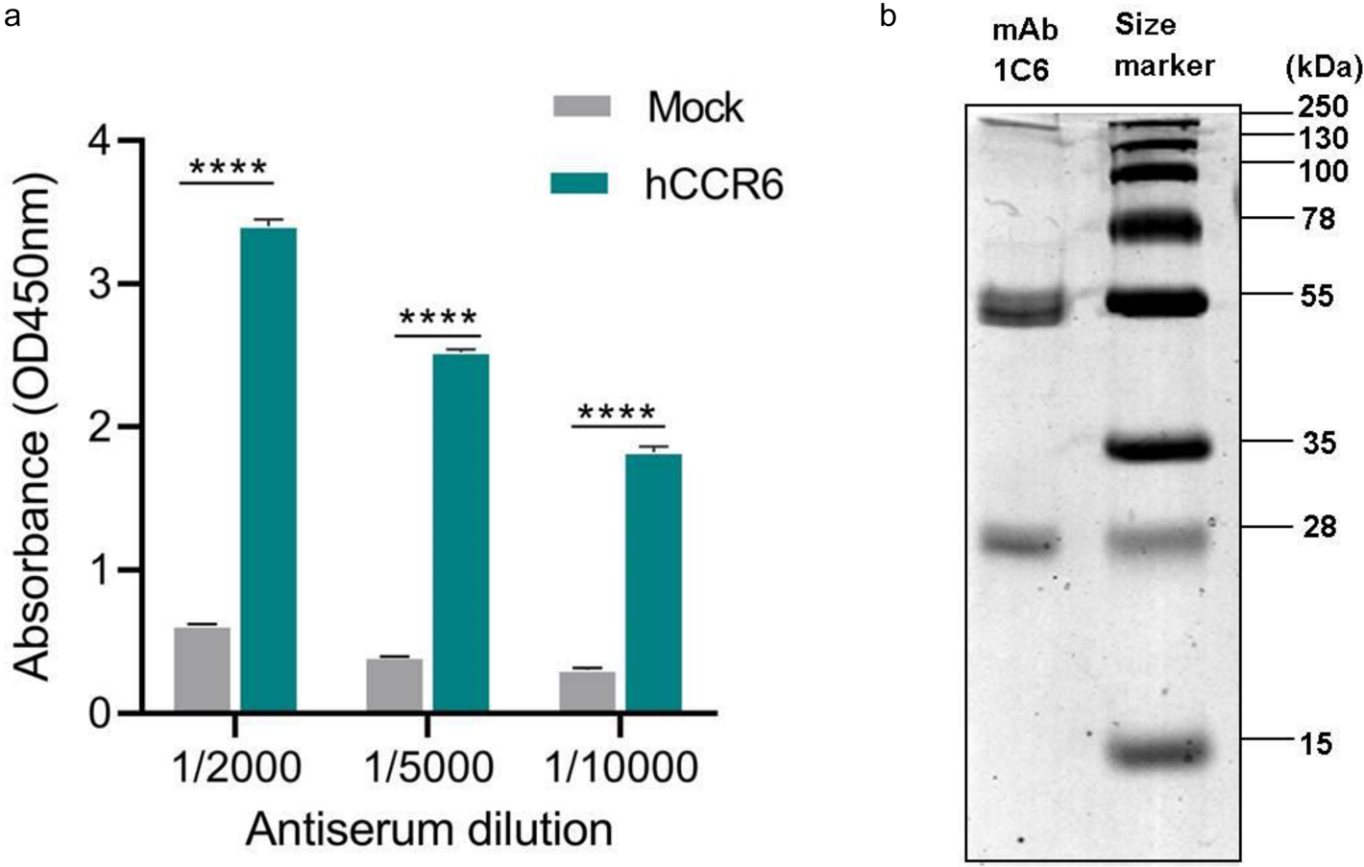
Establishment of anti-hCCR6 mAb. **a** Serum antibody titer of selected mouse after immunization with P815-c-myc-hCCR6 cells was tested in hCCR6-RBL and mock-RBL cell membrane ELISA. **b** SDS-PAGE analysis of 1C6 mAb purified from hybridoma supernatant using Protein G Sepharose. Positions of molecular size markers are shown on the right. **** *p* < 0.001

To generate antibodies against hCCR6, Balb/C mice were immunized with whole P815 cells which express high levels of transfected c-myc tagged hCCR6. Immunization of mice induced high serum titers of human CCR6 specific antibodies, as demonstrated by cell membrane ELISA. Serum antibodies were found to bind to cell membrane of RBL-HA-hCCR6 up to 1:10,000 dilutions, but not to cell membrane of mock-transfected RBL cells (Fig. 1A). Mouse with the highest hCCR6 specific antibody titer was sacrificed and a fusion was carried out. Approximately 679 hybridoma clones were generated by fusing NS1 myeloma cells with splenocytes from immunized mouse. Flow cytometry screening of hybridoma supernatants with HA-tagged hCCR6 RBL cells was used to remove the hybridomas that reacted with c-myc tag or P815 cells. Screening resulted in the identification of nine hybridoma candidates and, following by the limiting dilution step, one specific clone to hCCR6, 1C6, was obtained. The selected mAb 1C6 bound to hCCR6 cells but not to mock-transfected cells. These results indicate that mAb 1C6 was specific for the hCCR6 receptor protein and not for the c-myc-tag or the immunization P815 cells. Immunoglobulin was purified from the 1C6 hybridoma culture supernatant using Protein G Sepharose, and the purity and recovery of mAb 1C6 was analyzed by SDS-PAGE (Fig. 1B). The average yield of functional mAb 1C6 was approximately 2.2 mg from 100 mL of culture supernatant. The isotype of the mAb 1C6 was determined to be IgG1.

### 1C6 mAb specifically recognizes the hCCR6 without cross-reaction with other GPCRs

**Fig. 2:**
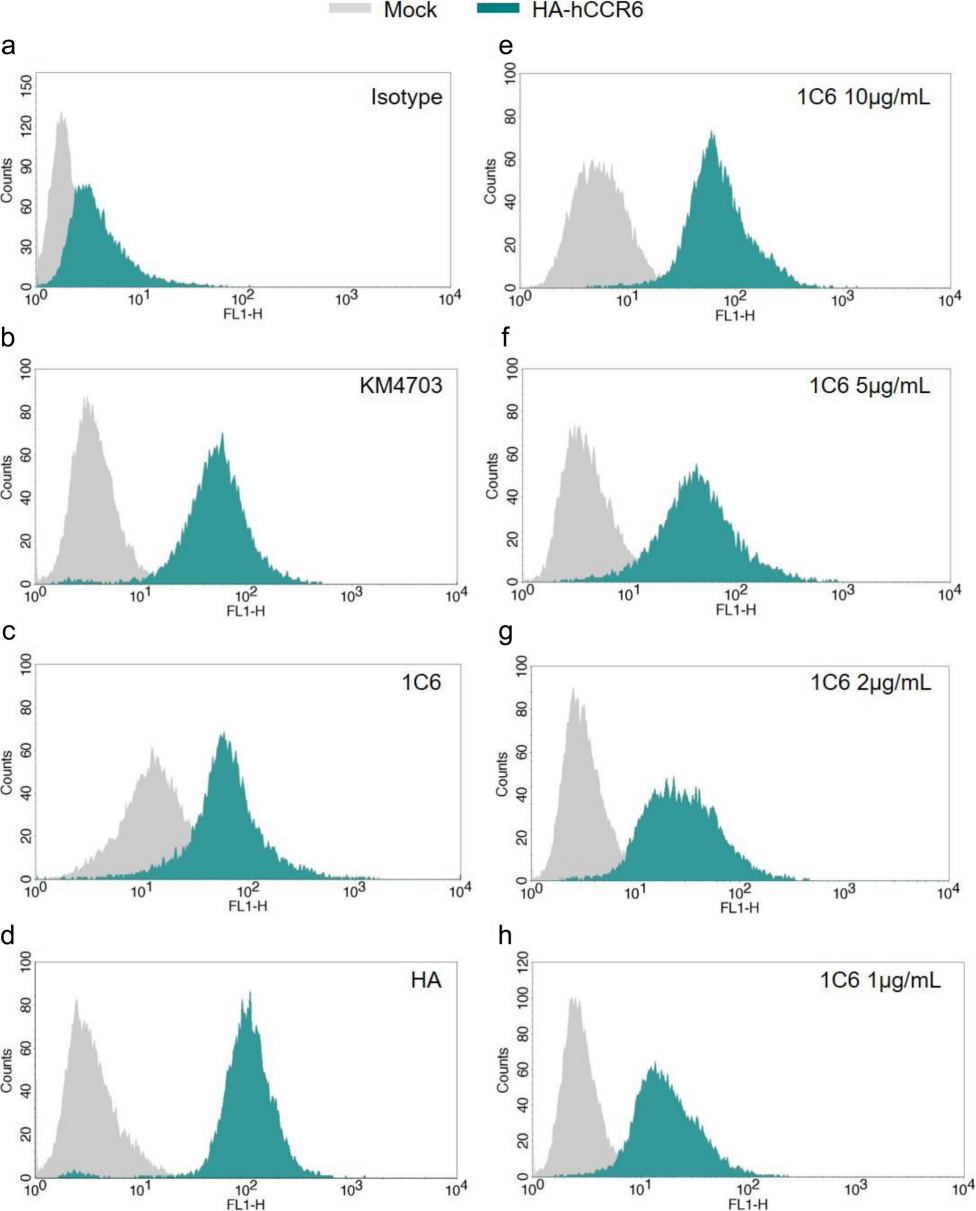
Binding of anti-hCCR6 mAb to RBL transfected cells with hCCR6. **a–d** Comparison of the reactivity against mock cells and HA-hCCR6-expressing RBL cells between control isotype **a**, KM4703 **b**, 1C6 **c** and HA **d** antibodies at 20 μg/mL. **e–h** Representative flow cytometry analysis of RBL-hCCR6 and mock cells stained with different doses of mAb 1C6 (1–10 μg/mL). Blue histograms of hCCR6^+^ cells are superimposed over grey histograms from mock transfectans. One representative analysis from three independent experiments is shown. The histogram overlays were performed using BD CellQuest Pro Software

To confirm the specificity observed during the screening procedure with hybridoma supernatants, we checked the ability of the purified mAb hCCR6 to recognize hCCR6 expressed at the surface of RBL or mock-transfected cells. The staining with the control isotype antibody, control KM4703 antibody, 1C6 antibody and control anti-HA antibody were compared (Fig. 2A–D). At the same mAb concentration (20 μg/mL) 1C6 showed 1.4-fold higher mean fluorescence intensity than KM4703 (MFI 97.3 vs. 68.8), but not stain mock transfectants. At lower concentrations (10 μg/mL, 5 μg/mL, 2 μg/mL and 1 μg/mL), 1C6 still stained more than 75% of hCCR6 cells (Fig. 2E–H), this indicates high mAb 1C6 affinity for hCCR6.

**Fig. 3:**
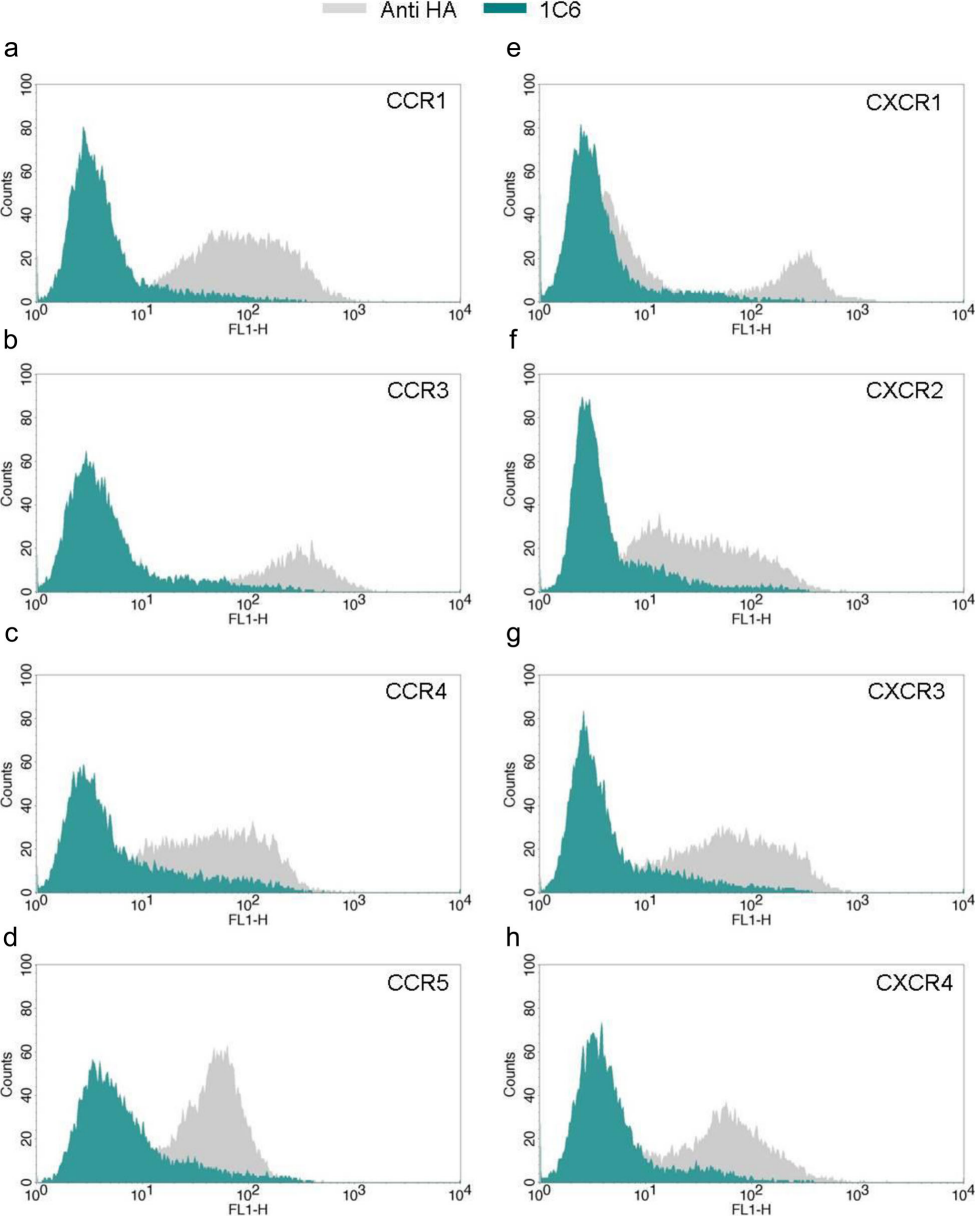
Crossreactivity of 1C6 mAb with other GPCRs. Stably RBL transfectants expressing hCCR1 **a**, hCCR3 **b**, hCCR4 **c**, hCCR5 **d**, hCXCR1 **e**, hCXCR2 **f**, hCXCR3 **g** or hCXCR4 **h** were stained with 1 μg/mL of 1C6 (blue histograms) or anti HA antibodies (grey histograms) and analyzed by flow cytometry. One representative analysis from three independent experiments is shown. The histogram overlays were performed using BD CellQuest Pro Software

Moreover, flow cytometry analyses revealed that mAb 1C6 did not recognize other human GPCRs. We observed that 1C6 did not cross-react with stable RBL transfectans expressing other humans GPCRs, including chemokine (C-C motif) receptors CCR1, CCR3, CCR4, CCR5 and chemokine (C-X-C motif) receptors CXC1R, CXC2R, CXC3R, CXC4R (Fig. 3), which have 25–45% identity with hCCR6. These data demonstrate that 1C6 is a specific mAb for hCCR6.

### The 1C6 mAb recognizes the N-terminal region of hCCR6

**Fig. 4:**
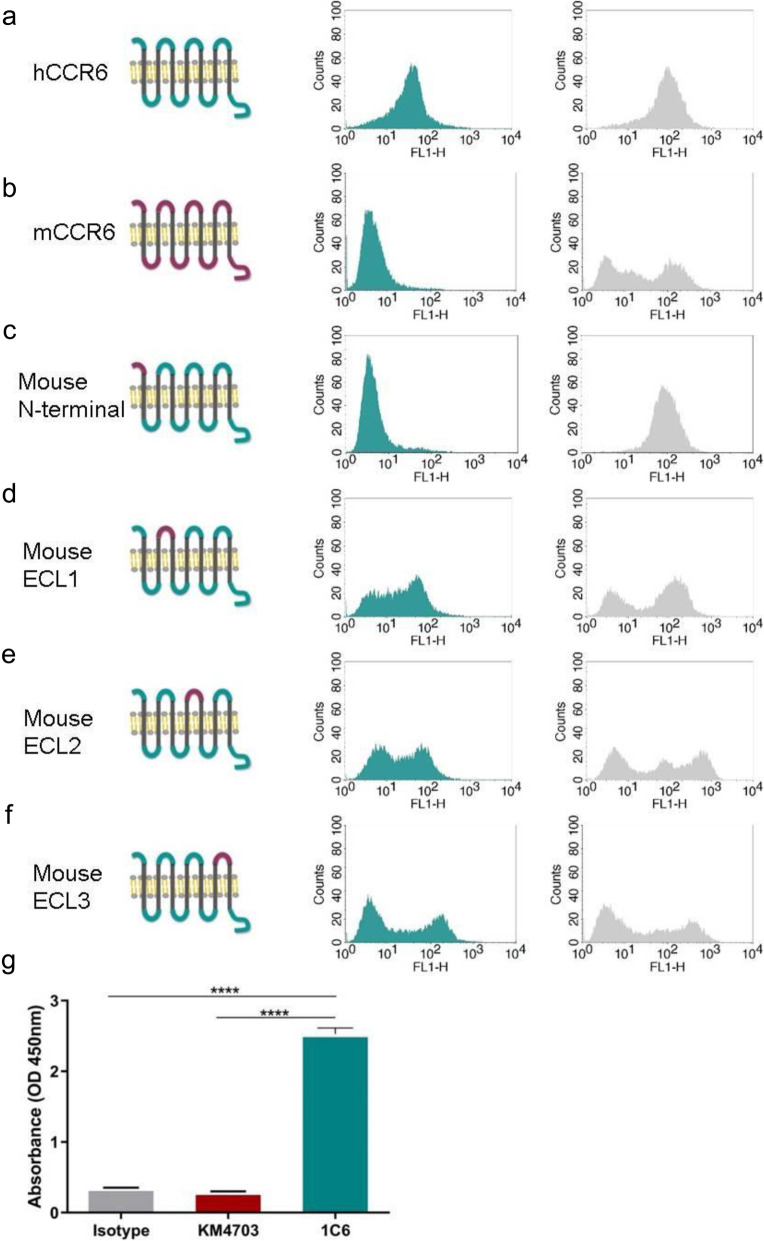
Eppitope mapping. **a–f** Extracellular domains and N-terminal of hCCR6 were swapped with the homologous region of mCCR6. Flow cytometric analysis was performed using 1 μg/mL of mAb 1C6 (blue histograms) or anti-HA (grey histograms) for staining hCCR6 **a**, mCCR6 **b** or swapped hCCR6 mutants **c–f** expressing HA tagged RBL cells. One representative analysis from three independent experiments is shown. **g** Binding of 0.5 μg/mL of isotype, KM4703 and 1C6 antibodies to human CCR6 N-terminal region by ELISA. Means and SD of triplicates are plotted. **** *p* < 0.001

Although human and mouse CCR6 share only 26% amino acid sequence identity (264 amino acid substitutions out of a total of 356 amino acid residues), the possibility that the mAb 1C6 recognized mouse CCR6 still remained. We showed, using mCCR6-stably transfected RBL cells, that the mAb 1C6 only recognized cells expressing human CCR6 and we observed no appreciable binding to cells expressing murine CCR6 (Fig. 4B).

To determine the epitope of mAb 1C6, we generated loop-swapped mutants of hCCR6 and stably transfected RBL cells with them, in which one of the three extracellular loops (ECLs) or N-terminus was changed to the corresponding region of mCCR6. The identity of ECLs and N-terminus for hCCR6 to mCCR6 was as follows: N-terminus (M1 to L47), 38%; ECL1 (S105 to K119), 66%; ECL2 (S181 to K211), 65%; and ECL3 (N280 to V303), 54%. As shown in Fig. 4 replacement of ECL1, ECL2 or ECL3 of hCCR6 did not affect the binding of mAb 1C6 to the chimeric CCR6, whereas replacement of the N-terminus of hCCR6 with mCCR6 resulted in loss of the binding of mAb 1C6 to hCCR6 (Fig. 4C), indicating that the epitope of 1C6 included N-terminal domain.

These results were confirmed by ELISA against peptide to the N-terminal (aa 1–47) region of hCCR6. When compared 0.5 μg/mL of KM4703 and 1C6 antibodies, we observed that KM4703 not recognized N-terminal peptide while mAb 1C6 bound to N-terminal region (Fig. 4G).

### Functional characterization of 1C6 antibody

For functional characterization of antibody 1C6 potency to inhibit the activation of hCCR6, β-arrestin recruitment and calcium flux were performed.

**Fig. 5:**
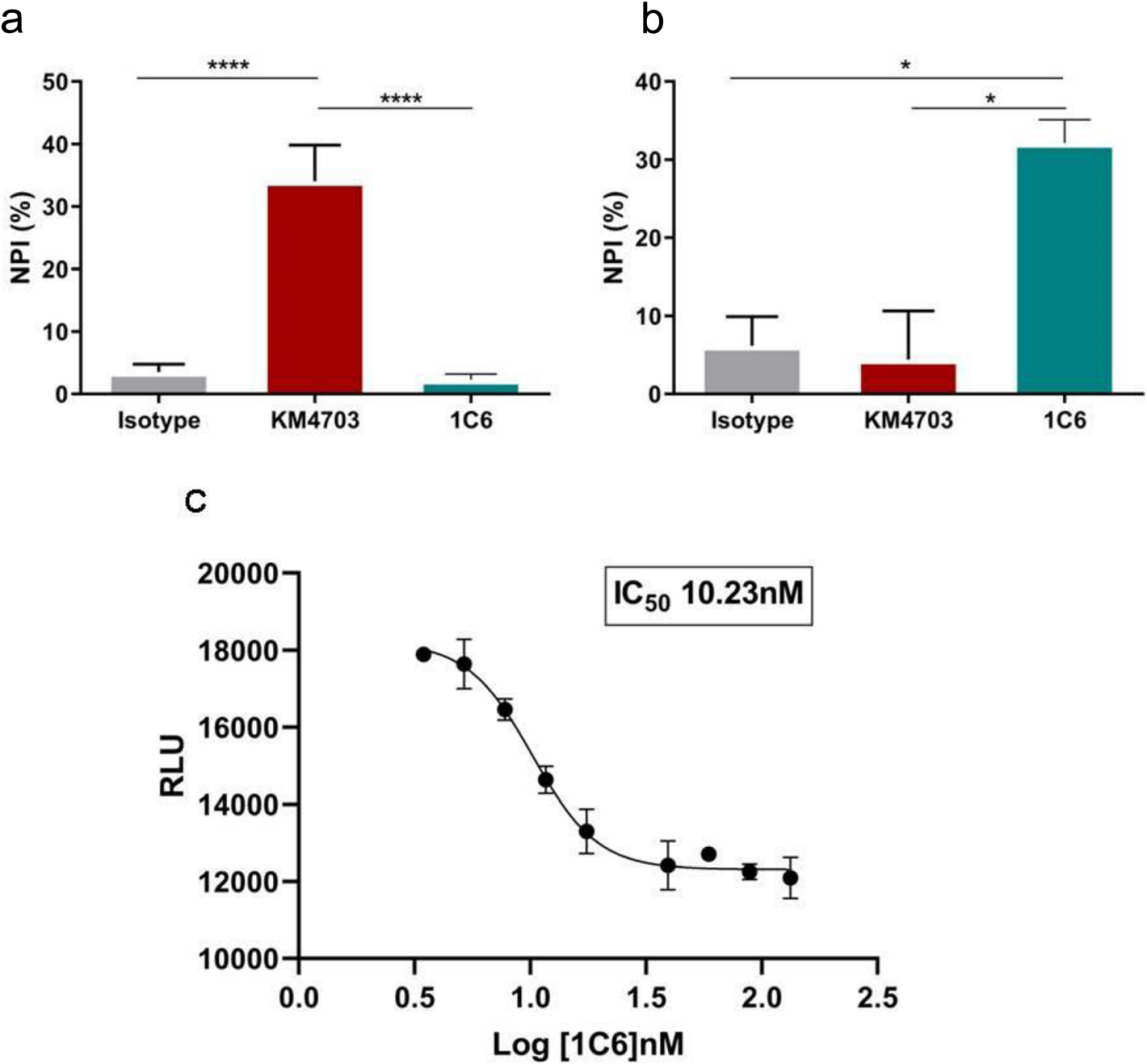
Characterization of mAB 1C6 effect on ligand-induced signaling. **a**, **b** Antagonistic potency of 20 μg/mL 1C6, KM4703 and mouse IgG1 was assessed against 10 nM of CCL20 on calcium flux **a** and β-arrestin **b** assays. Data were expressed in terms of normalized percent inhibition (NPI). **c** Assessment of inhibition was carried out in the presence of increasing concentrations of purified 1C6 antibody. Data from β-arrestin assay were expressed in terms of relative luminescence units (RLU). Means and SD of triplicates are plotted. * *p* < 0.05; **** *p* < 0.001

First, the capacity of the mAb 1C6 to inhibit hCCL20-mediated Ca^2+^ flux in CCR6-expressing cells was determined. The addition of mAb 1C6, even at a concentration of 20 μg/mL, did not reduce a Ca^2+^ flux in hCCR6-RBL cells (Fig. 5A), showing that mAb 1C6 did not have antagonistic activity on calcium signal. In contrast, KM4703 anti-hCCR6 control, at a concentration of 20 μg/mL, was an inhibitor of Ca^2+^ response.

β-arrestin recruitment was also measured with PathHunter β-arrestin GPCR assay system. In the antagonist assay, when compared the effect of mAbs at 20 μg/mL we observed that mAb 1C6 showed 32% inhibition of signal, however, KM4703 and isotype showed minimal inhibition of CCL20 response (Fig. 5B). These results suggest that mAb 1C6 had antagonistic activity specifically for hCCR6 in this assay. In addition, the capacity of mAb 1C6 to inhibit the CCL20 induced β-arrestin recruitment was also determined at various concentrations. At shown in Fig. 5C, mAb 1C6 inhibited CCL20-induced arrestin recruitment in a dose-dependent manner, with IC_50_ value of 10.23 nM. These results indicate that mAb 1C6 has antagonist effect via β-arrestin.

### 1C6 Inhibits the chemotaxis of CCR6^+^ cells

**Fig. 6:**
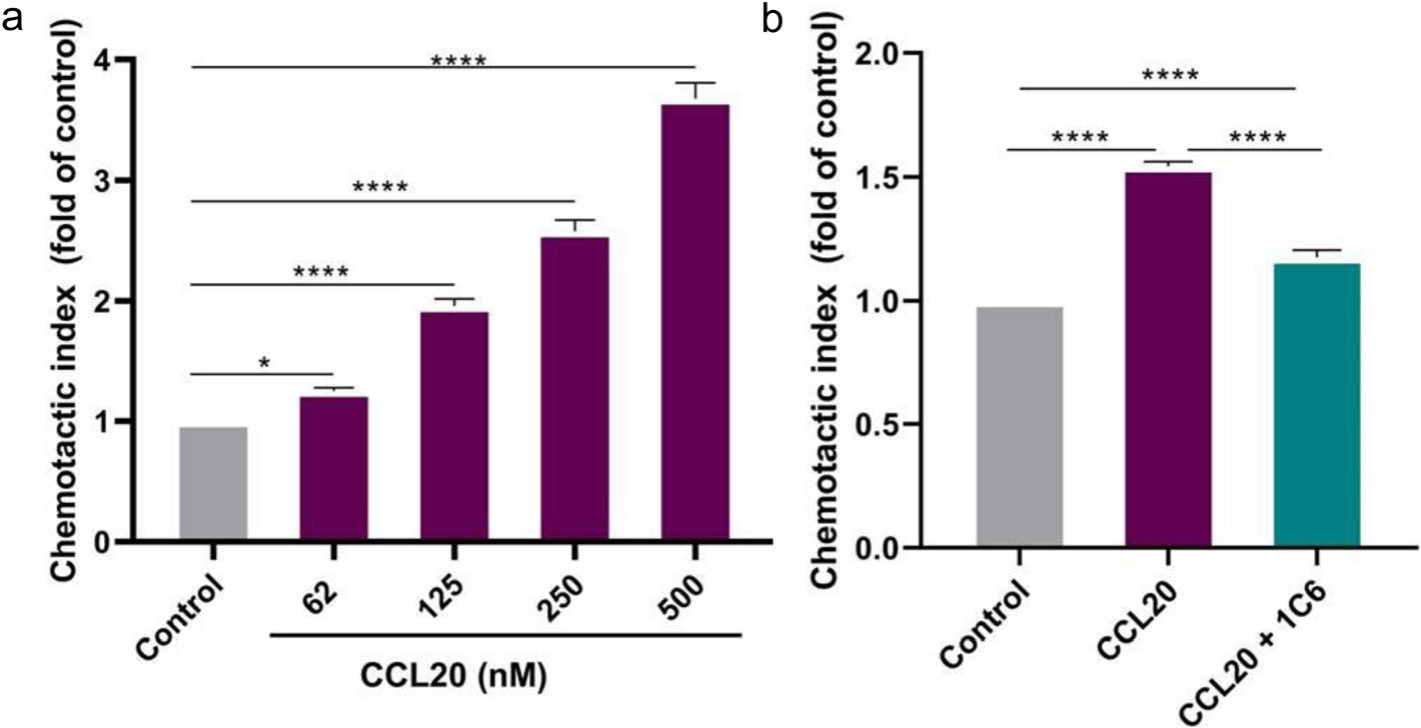
Inhibition of CCL20 mediated chemotaxis by 1C6 antibody. **a** Transwell chemotaxis assay on RBL-HA-CCR6 cells in the presence of increasing concentration of CCL20. The migrated cells were analyzed at 22 h of culture. **b** Assessment of hCCR6 cells chemotactic response to 125 nM CCL20 was carried out in presence or absence of 20 μg/mL 1C6. The chemotaxis index was calculated using the cells migrated in presence of CCL20 by the cells migrated in absence of CCL20 (control). All data are expressed as the mean ± SD of triplicated samples. * *p* < 0.05; **** *p* < 0.001

To determine the ability of 1C6 to block the migratory response of RBL-HA-hCCR6 cells to CCL20, chemotaxis experiments were performed. In order to quantify the cell migration ability to the hCCR6 ligand, cells were assayed with different concentration of CCL20 showing significant migration to CCL20 in a dose-dependent manner (Fig. 6A). The effect of 1C6 on transfected overexpressing hCCR6 cells migration in response to CCL20 was investigated and a decrease of chemotactic index was detected. As shown in Fig. 6B the 1C6 antibody inhibits ligand-induced chemotaxis, decreasing 68% of cell migration to ligand at this chemokine concentration.

### 1C6 reduce IL-17A expression by Th17 cells but not affect CCL20 production

**Fig. 7:**
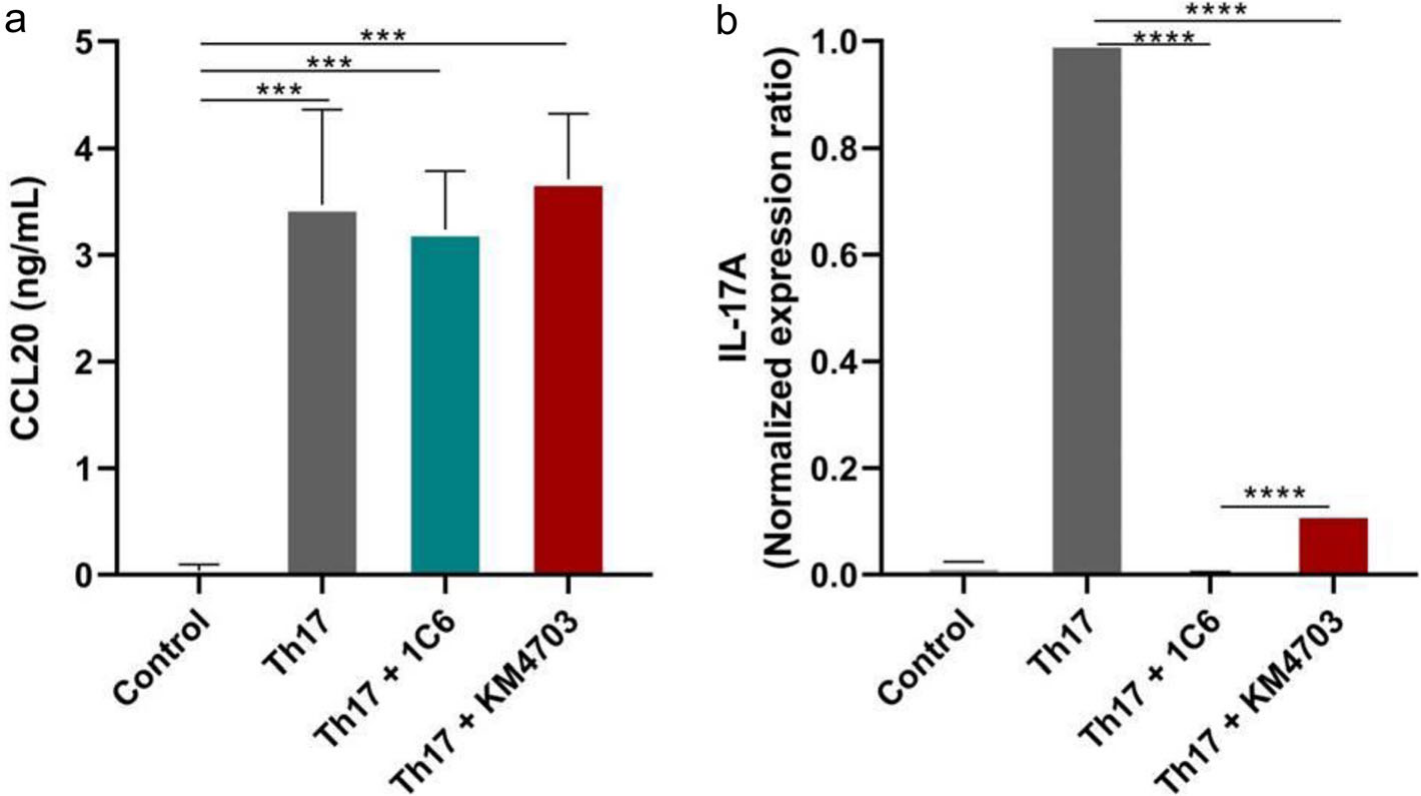
Effect of 1C6 antibody on Th17 cells. Human Th17 cells or control cells without Th17 polarizing conditions were culture with anti-CCR6 antibodies for 5 days. **a** The concentration of CCL20 in the cell culture supernatants was measured by ELISA. Means and SD of triplicates are plotted. *** *p* < 0.01. **b** The mRNA expression of IL-17A was measured by real-time PCR. Data were normalized to the reference gene β-actin and the normalized expression ratio was calculated using 2^-ΔΔCt^ method. Means and SD of duplicates are plotted. **** *p* < 0.001

The expression of CCL20, the only known ligand for CCR6, was analyzed by/in ELISA. In Th17 cells, CCL20, was produced a 3 ng/mL and there was no production in undifferentiated control T cells. The treatment of Th17 cells with 1C6 and KM4706 antibodies did not affect CCL20 production (Fig. 7A).

Effects of anti-CCR6 antibodies on IL-17A expression by human Th17 cells or undifferentiated control cells were determined by real-time PCR. IL-17A mRNA was specifically expressed in Th17 cells, which indicated proper differentiation of Th17 cells in vitro. To determine the effect of anti-CCR6 antibodies on IL-17A expression, human Th17 cells or undifferentiated control cells were culture with 1C6 or KM4703 antibodies for up to 5 days and then analyzed IL-17A mRNA expression. Although KM4703 decreased the expression of IL-17A, the antibody 1C6 showed strong response and completely abolished IL-17A expression (Fig. 7B).

## Discussion

Homeostasis disruption of CCR6/CCL20 axis plays a critical role in inflammation and autoimmune diseases, suggesting the potential of CCR6 as therapeutic target for patient treatment [5, 14, 15]. Antagonism of CCL20 binding to hCCR6 provides a suitable strategy to reduce symptoms of Th17-type inflammatory diseases [16, 17]. In the study reported here, we describe the generation and characterization of a specific anti-human CCR6 antibody with antagonistic activity.

When attempting to generate monoclonal antibodies capable of recognizing the native protein, it is also critical to use the target protein in its native conformation, not only in the immunization step, but also for the screening procedure [18]. Immunization with the purified form of the native receptors is not feasible due to the technically difficult to obtain this immunogen [19]. Only a few residues of extracellular loops and N-terminal domain are exposed as potentially immunogenic regions but the immunization of mice with peptides sequences of extracellular domains failed to induce antibodies reactive against native CCR6. Therefore, obtain monoclonal antibodies with functional activity remains a challenge. There are multiple antigen formats for immunization such a DNA, membranes, whole cells, virus-like particles (VLP), proteoliposomes, peptides and lipid nanodiscs [19–21]. Whole cells over-expressing GPCRs on the surface may be used as a useful immunogen because this method of immunization has the advantage that the over-expressing cell line presents the GPCR to the immune system in its native conformation and provides the highest level of stability. Moreover, this approach allows immunize with an antigen syngeneic expressing cell, which is more closely matched genetically to the host animal, maximizing specific immune response to the target membrane protein [22].

To generate hCCR6 antibody, we applied whole cells immunization with cell line overexpressing human CCR6 receptor that maintaining the conformational structure of hCCR6. Using these cells as antigen, we successfully obtained a functional antibody, 1C6, against hCCR6. Dreyer et al. reported a relation in the specific clones obtained in a fusion with elevated levels of target antigen expression [18]. Probably our results are related with the high level of CCR6 expression in our immunization cell line. Therefore, whole cells overexpressing target immunization could be a powerful approach to generate specific monoclonal antibodies against CCR6, and maybe against others GPCRs.

Although substantial progress has been made in generating therapeutic mAb candidates against a broad range of GPCR targets [23] the required specificity and selectivity is a problem for receptors in which drugs interfering with more receptors that the interest receptor [8]. Small molecules are known to be less selective and, generally, have less efficacy and potency than monoclonal antibodies [24]. The antibody 1C6 generated here was highly specific for human receptor, did not recognize the murine receptor and flow cytometry experiments, using stably transfected cells with other human chemokine receptors, showed that did not cross-react with related chemokine GPCRs. This unique specificity is an important advantage against small molecules, because they often bind non-specifically to other family members of GPCRs [8]. These data demonstrate that immunization using transfected cells with high levels of GPCR expression can be a useful strategy to obtain antibodies specific for the chemokine receptor target.

The N-terminal domain has a critical role for ligand recognition and activation of most GPCRs. Several authors suggested that the development of drugs or therapeutic antibodies against N-terminal, which have their activity blocking the chemokine binding, have potential as therapeutic tools [25, 26]. As shown with chimeric transfectants in flow cytometry and ELISA, 1C6 antibody mapped to an epitope located in the human CCR6 N-terminal domain. Because molecular characterization of CCR6 demonstrates that N-terminal domain is essential for ligand binding and signal transduction [27], it was interesting to study whether our antibody, that binds to N-terminal domain, was could inhibit any signaling pathway mediated by the binding of CCL20 to CCR6.

Binding of CCL20 to hCCR6 elicit a combination of responses, including activation of heterotrimeric G proteins and β-arrestin mediated signal transduction. Arrestins and G proteins have different non-overlapping functions and play important roles in GPCRs signaling [28]. G protein dependent mechanism result in GDP to GTP exchange on the Gαi subunit and triggers the activation of second messenger signaling and multiple intracellular pathways such a cyclic adenosine 3′, 5′ monophosphate (cAMP), inositol triphosphate (IP3), Ca^2+^, diacylglycerol (DAG), mitogen-activated protein kinase (MAP kinase) and Rho/Rac [29]. Whereas arrestins mechanism involve the GPCRs phosphorylation by GPCR kinases (GRK), which trigger the recruitment of β-arrestins causing GPCR desensitization and internalization, initiating a G-protein independent wave of downstream signaling [30]. If a functional assay capturing only one signaling pathway is selected for screening neutralizing activity of our antibody, potentially inhibitory activity could be no detected if the antibody does display biased activity. Therefore, antagonistic activity of our antibody may be elucidated using arrestin and G protein dependents pathways. Thus, we characterized the antagonism of 1C6 antibody in β arrestin and calcium flux assays. Although 1C6 showed no antagonistic effect in calcium signaling pathway at concentration up to 20 μg/mL (133 nM), it did have an antagonistic effect in β-arrestin recruitment assay (IC_50_ 10.23 nM). Therefore, we demonstrated inhibition of CCL20 induced signaling on β-arrestin recruitment but not on calcium flux assay. These results could be due to the activity of our antibody differs in dependence on signaling pathway. One molecule can act as agonist, partial agonist or antagonist according to the signaling pathway investigated [31]. Biased agonists display better efficacies in activating one pathway over others and screening of these compounds is a critical issue because assays based on one signaling pathway might miss potentially valuable compounds acting on other pathways [1]. These examples show that biased ligands can have markedly different signaling properties compared to the endogenous ligand which activates both pathways. Is known that activation of different signaling pathways in GPCRs may have opposite effects and this biased signaling can be applied in the development of novel therapies [32, 33]. β-adrenergic receptor is a paradigm of this fact and many other examples has been reviewed [34]. CCR-chemokine receptors CCR2, CCR5, CCR7, CCR10 displayed significant bias in terms of G protein activation and β-arrestin recruitment for their ligands CCL8, CCL5, CCL19/ CCL27 and CCL28, respectively. There are partial or biased agonist of chemokine receptors, which are characterized by a selective loss of efficacy in certain types of signaling [35]. Biased allosteric modulators offer the possibility of designing, developing, and producing safer, more targeted pharmacologic therapies [36]. Biased signaling pathways have been described for some specific GPCRs such as angiotensin and β-adrenergic receptors. A drug described as biased antagonist is losartan, an angiotensin II antagonist, that was found to be a G-protein biased antagonist, leaving β-arrestin signaling largely unaffected [37]. We obtained a biased antagonist which has neutralizing activity on arrestin signaling, but not on calcium flux, and function as potent inhibitor of this pathway. The control antibody KM4703 did not bind to N-terminal region and not inhibit the arrestin signaling, but was able to block calcium flux pathway. Dorgham et al., obtained mAb that bind to N-terminal and did not block calcium flux signal but arrestin via was not assayed [38]. Taken the results together, we hypothesized that the antagonistic activity via N-terminal domain is related with arresting signaling but not with the calcium flux signaling.

Th17 cells are a subset of T lymphocytes and their differentiation is mediated by TGF-β and IL-6 signaling. Th17 cells provoke a wide range of inflammatory reactions via their specific cytokines IL-17 and IL-22. It has been reported that hCCR6 is expressed on surface of Th17 cells and secrete CCL20 promoting the recruitment of another Th17 cells to the sites of the inflammation [3]. Our antibody blocks the migration of CCR6 expressing cells to CCL20 and could be act inhibiting the recruitment of Th17 cells to inflammatory tissues. Based on the inhibition of chemotaxis of CCR6 expressing cells to CCL20 showed in this study, 1C6 antibody could be a good candidate for suppress the recruitment of Th17 cells to inflamed tissues, although further studies with Th17 cells are needed to confirm our hypothesis.

IL-17A is a pro-inflammatory cytokine that plays an essential role in inflammatory response and elevated levels of this cytokine have been associated with the development of immune-inflammatory diseases [39]. In our study we demonstrate that 1C6 antibody not affects to CCL20 production by Th17 cells, however the expression of IL-17A on these cells was abolished by our antibody. We observed, higher reduction of IL-17A expression with 1C6 than KM4703 antibody, these differences in expression inhibition it could be due to antibodies blocks different signaling pathways. Although further research is needed to demonstrate the molecular connection between CCR6 expression and IL17 production, it has been demonstrated the association between the ability to produce IL-17 and the expression of CCR6 by human T cells [40]. In addition, it has been described that decrease of β-arrestin levels reduce expression of Th17-associated cytokines, such as IL-17 [41]. While antibody 1C6 inhibits arrestin pathway, antibody KM4703 inhibits G protein dependent pathway and it could be indicating that inhibition of arrestin pathway produces a greater response that inhibition of G protein dependent pathways. IL-17 inhibitors are a new and promising therapeutic option for treatment of inflammatory diseases and three anti-IL17 monoclonal antibodies, secukinumab, brodalumab and ixekizumab, are approved for psoriasis treatment [42]. Based on the results obtained, 1C6 antibody it could be an alternative to treatments with anti-IL-17.

There are currently only two monoclonal antibodies against GPCRs, mogamulizumab and erenumab which target CCR4 and CGRP type 1 receptor respectively, approved by the Food and Drugs Administration (FDA) [7, 26, 43]. Around sixteen CCR6-CCL20 inhibitors have been investigated but to the date no therapeutic agents targeting CCR6 have progressed into clinical evaluation [5]. The company ChemoCentryx has developed a small molecule (CCX9664) against CCR6 but they have not yet released clinical results. Unlike others GPCRs, this target has proven difficult in small molecules screen and other therapeutics approaches, like the development of blocking antibodies, have to be explored [44]. In summary, the antibody reported here represents a potential therapeutic opportunity.

## Conclusion

In the present report we generated a monoclonal antibody, 1C6, which bound to N-terminal domain of hCCR6. 1C6 antibody was shown to be suitable for flow cytometric detection and was shown CCR6 biased and selective antagonistic activity on arresting signaling. Moreover, the chemotaxis of CCR6 expressing cells and the expression of IL-17A on Th17 cells were reduced with 1C6 antibody. Therefore, 1C6 will be a helpful pharmacological tool for elucidating the biological effects of CCL20 in various mouse model systems and should become useful in vitro and in vivo studies of hCCR6-mediated diseases.

We demonstrated that whole cells immunization is an efficient and effective approach to generate specific and selective monoclonal antibodies against hCCR6 and a similar approach could be applied to other GPCRs targets and could drive the discovery of selective GPCR antibodies with high therapeutic potential.

In conclusion, the results described here demonstrate that the generated 1C6 antibody blocks functional activity of ligand-induced CCR6-mediated signaling. Therefore, 1C6 has therapeutic potential for the targeted neutralization of CCR6^+^ cells and could be used as treatment of inflammatory diseases alone or in combination with other therapies.

## Supplementary Information

**Additional file 1.**

## Data Availability

The datasets used and analyzed during the current study are available from the corresponding author on reasonable request.

## Abbreviations

mAb: Monoclonal antibody
hCCR6: Human CC chemokine receptor 6
GPCRs: G-protein-coupled receptors
CCL20: CC chemokine ligand 20
Th17: T helper 17
Treg: T regulatory
ELISA: Enzyme-linked immunosorbent assay
FBS: Fetal bovine serum
BSA: Bovine serum albumin
