# Supplementary figures and images for: Amino terminal recognition by a CCR6 chemokine receptor antibody blocks CCL20 signaling and IL-17 expression via β-arrestin
Source: BMC Biotechnol. 2021 Jul 5;21:41. doi: 10.1186/s12896-021-00699-2 (PMC8259436; doi:10.1186/s12896-021-00699-2)

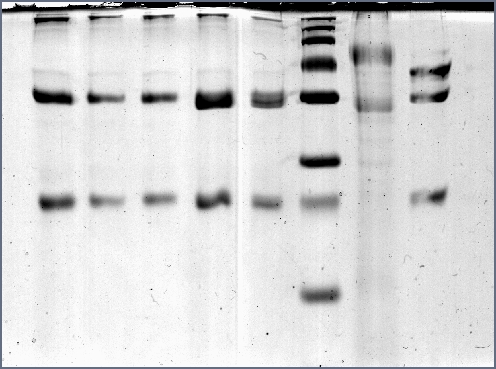

Supplement: Supplementary file 1 — Additional file 1. [file 12896_2021_699_MOESM1_ESM.jpg]
